# Dietary Vitamin C and Age-Induced Lipid and Hormonal Metabolic Changes in a Humanized Mouse Model Not Synthesizing Vitamin C and Producing Lipoprotein(a) [Gulo (−/−); Lp(a)+]

**DOI:** 10.1155/2021/5591697

**Published:** 2021-06-15

**Authors:** Lei Shi, Matthias Rath, Aleksandra Niedzwiecki

**Affiliations:** Dr. Rath Research Institute, 5941 Optical Ct, San Jose, California 95138, USA

## Abstract

The lack of ability to produce vitamin C innately and the ability to synthesize human lipoprotein(a) (Lp(a)) are two unique metabolic features present in humans, compared with most other animal species. The Gulo (-/-) and Lp(a)+ mouse model displays these two features and is therefore suitable for the study of metabolic aspects relevant to human metabolism. It is a well-known fact that vitamin C is essential in collagen synthesis, and in maintaining extracellular matrix integrity, as well as being a powerful antioxidant and cofactor in many metabolic pathways, which makes it a critically important micronutrient for health and healthy aging. In this study, we investigated the effects of a long-term intake of high and low doses of vitamin C on age-related metabolic lipid and hormonal changes in young (eight to nine months), mid-aged (one year), and old (two years) Gulo (−/−) and Lp(a)+ mice. We observed that chronic vitamin C deficiency resulted in a less healthy metabolic lipid profile, impaired serum insulin-like growth factor (IGF-1), and sex-hormones secretion, all of which can accelerate the development of various pathological conditions in the aging process. The most susceptible to the negative impact of vitamin C deficiency were the young (eight to nine months) and old (two years) mice. Our study conducted in this humanized mouse model indicates that sustained adequate vitamin C intake is essential in maintaining a healthier metabolic profile, important in preventing age-related pathologies throughout the aging process.

## 1. Introduction

One of the distinct features of human metabolism compared with about 99% of other animals is the lack of internal vitamin C synthesis and the production of human lipoprotein(a).

Humans and a few animal species, including nonhuman primates and guinea pigs, are not able to manufacture vitamin C internally, owing to a loss of gene coding for L-gulonolactone oxidase. At the same time, humans, unlike the majority of animals, with only few exceptions (primates, guinea pigs, and hedgehogs) can synthesize lipoprotein(a) (Lp(a)). Lp(a), a variant of low-density lipoprotein (LDL), has been associated with the development of coronary heart disease and proven to be an atherosclerosis risk factor [1]. The Lp(a) molecule contains LDL, linked by a disulfide bridge to a large protein, apolipoprotein(a) (apo(a)), making it more adhesive, and its vascular deposition parallels the progression of atherosclerosis [2, 3].

These two unique aspects of human metabolism appear related, since internal production of Lp(a), which occurred about 60 million years ago in our primate ancestors, coincided with a loss of the ability to synthesize vitamin C innately. In the most rational explanation of these overlapping genetic events, Rath and Pauling proposed [4] that Lp(a) functions as a physiological surrogate for vitamin C. It aims primarily at protecting the integrity of the vascular wall compromised by vitamin C deficiency, through its vascular deposition. In this function, vascular deposits could prevent the deadly consequences of blood loss from scurvy [4]. However, with sustained long-term vitamin C deficiency, various pathological conditions develop, leading to atherosclerosis, further aggravated by the prothrombotic and proinflammatory effects of Lp(a) [5, 6].

With rare Lp(a) representation in the animal world, the animal models for the study of Lp(a) metabolism and pathogenicity are scarce, mostly limited to transgenic rabbit and mouse models [7, 8]. Experimental studies on vitamin C in various aspects related to human metabolism have been largely conducted on guinea pigs and a recently developed mouse model lacking gulonolactone oxidase activity, Gulo (−/−) [9–11]. To our knowledge, only the transgenic mouse model, Gulo (−/−) and Lp(a)+, that we developed combines these two important characteristics of human metabolism in one organism. This mouse model lacks the Gulo gene; thus, it does not synthesize vitamin C, and at the same time, it carries two mutations expressing human apolipoprotein(a) (h-apo(a)) and human apolipoprotein(B) (h-apo(B)) from human Lp(a).

This mouse model has been applied in studying various aspects of heart disease, cancer, and diabetes [5, 6, 12]. Our earlier study in Gulo (−/−) and Lp(a)+ mice documented that, after six weeks of vitamin C deficiency, the serum Lp(a) levels increase, which correlates with increased Lp(a) deposition on the structurally impaired vascular walls and atherosclerosis [5]. Most recently, we documented that a long-term insufficient intake of vitamin C results in age-related increased deposition of Lp(a) in the brain's blood vessels in aging Gulo (−/−) and Lp(a)+ mice [13].

Vitamin C has multiple functions, including its critical role in collagen synthesis, antioxidant protection, and anti-inflammatory and modulating lipid metabolism effects, making it an important micronutrient in healthy human aging [14]. It also plays an important role in age-related changes in the endocrine system, affecting insulin growth factor 1 (IGF-1) and sex steroids, among others. Higher intake of dietary vitamin C was found to be associated with higher concentrations of serum IGF-1 in healthy women, which reduced the risk of occurrence of some chronic diseases [15]. Vitamin C also modulates the estrogen synthase activity in rats [16], and it increases the plasma estrogen levels in menopausal women during hormone therapy [17].

Surprisingly, even in industrialized countries, vitamin C deficiency has been widely recorded in people at all age ranges [18, 19]. For example, a study conducted in 2004 found that 10% of men and 7% of women aged over 20 exhibited vitamin C deficiency [20]. Furthermore, vitamin C deficiency is more prevalent in older populations [21, 22]. Because the older population has higher requirements for vitamin C, it is particularly important for older adults to obtain an adequate daily vitamin C intake [23, 24].

To our knowledge, there is limited information on the effects of a long-term intake of vitamin C on metabolic changes in relation to cardiovascular risk factors and hormonal status during aging, especially when tested on adequate models resembling human metabolism. The Gulo (−/−) and Lp(a)+ mouse is a unique model to study the effects of dietary vitamin C in a human-like metabolism. In this study, we investigated the comprehensive effects of a long-term high and low vitamin C intake on the lipid profile and on sex- and age-related hormonal changes during the aging process.

## 2. Materials and Methods

### 2.1. Animals

Human Gulo (−/−) and Lp(a)+ mice were generated as described previously [5]. Briefly, homozygous Gulo (−/−) mice were first generated from breeding heterozygous Gulo(±) mice BALB/cBy-Gulo^sfx^/J (Jackson Laboratory, Sacramento, CA). Then, Gulo (−/−) and h-apo(a)+ mice and Gulo (−/−) and h-apoB-100+ mice were generated from separately breeding homozygous Gulo (−/−) mice with human apo(a) [h-apo(a)] transgenic mice (Mutant Mouse Regional Resource Center, Columbia, MO) and human apoB-100 (h-apoB-100) transgenic mice (Taconic Farms Inc., Hudson, NY). These two transgenic mice were then bred to generate Gulo (−/−) and Lp(a)+ mice.

To ensure the homozygosity of the Gulo locus knockout and the presence of h-apo(a) and h-apo(B)-100 genes, genotyping was performed by TaqMan FAM probe Real Time-PCR at Transnetyx (Cordova, TN) using mouse tail clips. All animal experiments were conducted with humane and customary care and followed a protocol approved by the internal Institutional Animal Safety Review Committee. All mice were housed in a barrier facility with a 12-hour light/12-hour dark cycle with food and water *ad libitum*.

### 2.2. Study Design

Experiments were undertaken using both male and female Gulo (−/−) and Lp(a)+ mice. Three groups of mice were used: mice aged eight to nine months (32–36 weeks), one year (52 weeks), and two years (104–116 weeks) at the time of harvesting. Twelve mice of each gender were randomly assigned to each age group. In each age group, six mice were assigned to a high-vitamin-C (H-VC) supplemented diet, which contained a modified LabDiet® Laboratory Rodent Diet 5001 with 1000 PPM vitamin C and distilled water (no vitamin C added), and six mice were assigned to a low-vitamin-C (L-VC) supplemented diet, which contained LabDiet® Laboratory Rodent Diet 5001 with 30 mg/L vitamin C added in distilled water. The H-VC diet provides approximately 4 mg ascorbic acid daily, and the L-VC diet with 30 mg/L vitamin C in distilled water provides mice with approximately 0.12 mg ascorbic acid daily. The duration of the experimental diet was 20 weeks. After 20 weeks, mice were harvested for blood and tissues. Serum was collected from blood drawn via cardiac puncture. Mouse livers were collected and fast frozen in liquid nitrogen. Mouse serum and livers were stored in −80°C until use.

### 2.3. Serum and Liver Ascorbic Acid Measurement

Frozen mouse liver was weighed and homogenized in Millipore water. The homogenates were then centrifuged at 2000 rpm at 4°C for 20 mins. The liver supernatants and serum samples were used for ascorbic acid determination by Biovision Ferric Reducing Ascorbate Assay (FRASC) kit (Milpitas, CA). The ascorbic acid levels were expressed as nmole/mL for serum samples and nmole/mg liver weight for liver samples.

### 2.4. Serum Lipid Profile (Total Cholesterol, LDL, HDL, and Triglycerides)

Total cholesterol (TC), high density cholesterol (HDL-C), low density cholesterol (LDL-C), and triglyceride (TG) levels were determined by homogeneous enzymatic colorimetric assay performed at the Comparative Pathology Laboratory (CPL) at the University of California (Davis, CA).

### 2.5. Serum Hormone Levels

The serum levels of testosterone in male mice, 17*β*-estradiol in female mice, and free IGF-1 were determined by using the following commercially available kits respectively: Abcam Testosterone ELISA Kit (Cambridge, MA), Biovision Estradiol (Mouse) ELISA kit (Milpitas, CA), and Abcam Mouse IGF-1 ELISA Kit (Cambridge, MA). The assays were performed according to the manufacturers' manuals.

### 2.6. Statistical Analysis

All data are presented as means ± standard deviation. The variance among the experimental groups was analyzed by two-way ANOVA followed by Tukey's HSD (honest significant difference) post hoc test for pairwise comparisons between groups in the case of main effects and interaction effects. A *p* value of <0.05 was considered statistically significant. All statistical analyses were performed using Python (version 3.8.5). Correlation analysis between serum LDL and serum IGF-1 levels was performed by multiple linear regression analysis using Microsoft Excel.

## 3. Results

### 3.1. Serum and Liver Ascorbic Acid

**Figure 1:**
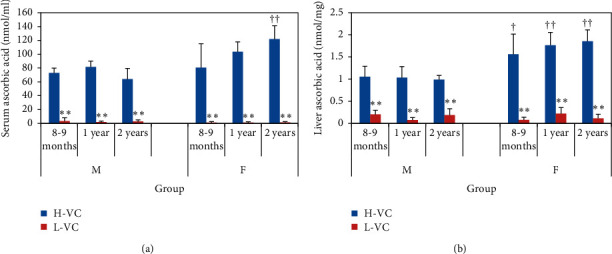
Serum ascorbic acid levels (a) and liver ascorbic acid levels (b) in each age group and gender. Data are expressed as mean ± SD. *n* = 3–6 mice per group. For Tukey's HSD tests, ^*∗∗*^represents statistically significant difference between H-VC groups and L-VC groups of the same age and gender at the significance level of 0.01; ^†^represents statistically significant difference between male and female mice of the same age and diet at the significance level of 0.05; ^††^represents *p* < 0.01.

Ascorbic acid levels in the serum and liver of Gulo (−/−) and Lp(a)+ mice fed for 20 weeks on an H-VC or L-VC diet are shown in Figure 1.

The serum ascorbic acid levels were significantly lower in L-VC mice in both genders and in three age groups compared with H-VC mice of the corresponding gender and age. As such, ascorbic acid levels in L-VC mice at the age of eight to nine months, one year, and two years in males were 3.3, 1.8, and 2.7 nmol/ml, respectively, and in females, they were 1.1, 1.2, and 1.5 nmol/ml, respectively (Figure 1(a); *p* < 0.01). Ascorbic acid levels in H-VC mice of each corresponding age in males were 72.9, 81.5, and 63.9 nmol/ml, respectively, and in females, they were 80.6, 103.6, and 121.9 nmol/ml, respectively.

In H-VC groups, serum ascorbic acid levels increased with age in female mice, reaching the highest level at the age of two years (Figure 1(a); *p* > 0.05). Furthermore, in H-VC groups, serum ascorbic acid levels were significantly higher in female mice than in male mice at the age of two years (Figure 1(a); *p* < 0.01).

Continuous intake of an L-VC diet for 20 weeks had almost depleted the ascorbic acid in the liver in mice of both genders at the age of eight to nine months, one year, and two years (male: 0.2, 0.1, and 0.2 nmol/mg, resp.; female: 0.1, 0.2, and 0.1 nmol/mg, resp.; Figure 1(b)) when compared with H-VC mice in each corresponding age (male:1.1, 1.0, and 1.0 nmol/mg, resp.; female: 1.6, 1.8, and 1.9 nmol/mg, resp.; *p* < 0.01). The liver ascorbic acid levels were consistently higher in females than males in all age groups (Figure 1(b); *p* < 0.05).

### 3.2. Serum Lipid Profile

Serum total cholesterol, LDL, HDL, triglycerides levels, and LDL/HDL ratios are shown in Table 1.

In H-VC groups, serum total cholesterol levels slightly increased in mice aged eight to nine months and one year and then decreased in two-year-old mice both in in males and females (*p* > 0.05). L-VC female mice had significantly lower total cholesterol levels than H-VC female mice at the age of one year (*p* < 0.05). Its level was significantly higher in L-VC male mice compared with H-VC male mice at the age of two years (*p* < 0.05).

Serum LDL levels in both H-VC male and female mice slightly increased at the ages of eight to nine months and one year and decreased in mice aged two years (*p* > 0.05). Compared with H-VC male mice, the LDL levels were significantly higher in L-VC males aged eight to nine months and two years (*p* < 0.05). Serum LDL levels in L-VC mice were lower than in H-VC mice aged one year in both genders (*p* > 0.05).

Serum HDL levels were similar in H-VC male mice aged eight to nine months and one year and significantly lower in two-year-olds (*p* < 0.05). In H-VC female mice, serum HDL levels significantly increased in mice aged eight to nine months and one year and decreased in mice aged two years (*p* < 0.05). Compared with H-VC male mice, serum HDL levels in L-VC males were significantly lower at the age of eight to nine months and one year (*p* < 0.05) but reached similar levels at the age of two years (*p* > 0.05). In L-VC female mice, the chronic vitamin C deficiency led to significantly decreased serum HDL levels in mice aged one year (*p* < 0.05) compared with H-VC mice but stayed at comparable levels in mice aged eight to nine months and two years (*p* > 0.05).

In mice of both genders in H-VC groups, there was a significant increase in triglyceride levels at the age of eight to nine months and one year and a subsequent decrease at the age of two years (*p* < 0.05). Compared with H-VC mice, serum triglyceride levels in L-VC mice were similar in both genders at the age of eight to nine months and one year (*p* > 0.05) and significantly higher in two-year-old animals (*p* < 0.05).

LDL/HDL ratio was significantly higher in L-VC male mice aged eight to nine months and two years than in H-VC male mice of corresponding ages (*p* < 0.01) and was similar in one-year-old males in both the L-VC and the H– VC groups (*p* > 0.05). In female mice, the LDL/HDL ratio did not differ between L-VC and H-VC groups at all ages (*p* > 0.05).

### 3.3. Serum Testosterone, Estradiol, and Free IGF-1

**Figure 2:**
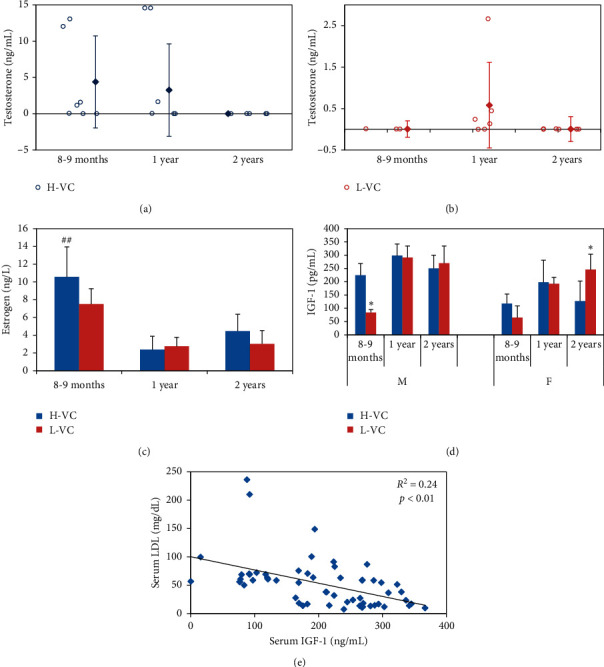
Serum levels of testosterone in H-VC male mice (a) and L-VC male mice (b), estradiol in female mice (c), free IGF-1 (d) in each age group and gender, and correlation between serum free IGF-1 and LDL (e). Data are expressed as mean ± SD. *n* = 3–6 mice per group. For Tukey's HSD tests, ^*∗*^represents statistically significant difference between H-VC groups and L-VC groups of the same age and gender at the significance level of 0.05; ^##^represents statistically significant difference between all three age groups of the same gender and diet at the significance level of 0.01.

Serum testosterone levels in H-VC male mice and L-VC male mice are shown in Figures 2(a) and 2(b), respectively. Serum testosterone levels in individual male mice on L-VC and H-VC diets are shown in Figures 2(a) and 2(b), respectively. Serum testosterone levels showed large individual variations, which are a common phenomenon in mice; therefore, in addition to calculated averages, we also present individual mice results. In H-VC groups, the average serum testosterone level was the highest in eight-to-nine-month-old mice (4.4 ng/mL), slightly decreased in one-year-old mice (3.3 ng/mL), and fell almost to zero in two-year-old mice. In L-VC mice, the average serum testosterone levels were much lower compared with H-VC groups. Young mice aged eight to nine months had a testosterone level of almost zero. Its level slightly increased to 0.6 ng/mL in one-year-old mice but was almost zero in two-year-olds.

Serum estradiol levels in female mice are shown in Figure 2(c). Estradiol remained at the highest level in eight-to-nine-month-old mice in both H-VC and L-VC groups compared with older mice. In H-VC groups, serum estradiol levels significantly decreased with age, from 10.6 ng/L in eight-to-nine-month-olds to 2.4 ng/L in one-year-olds and 4.5 ng/L in two-year-olds (*p* < 0.01). Compared with H-VC mice, serum estradiol level in L-VC mice was slightly lower in mice at the age of eight to nine months (10.6 ng/ml vs. 7.5 ng/L; *p* > 0.05) and remained at similar levels at the age of one year and two years (2.4 ng/ml vs. 2.8 and 4.5 ng/ml vs. 3 ng/L, respectively; *p* > 0.05).

Serum free IGF-1 levels presented in Figure 2(d) show that IGF-1 in H-VC groups significantly increased in both genders at the age of eight to nine months to one year and slightly decreased at the age of two years (male: 224.8, 298.3, and 250.3 pg/mL, resp.; female: 118.3, 198.5, and 127.3 pg/mL, resp.; *p* < 0.05).  They remained at similar levels in one-year-old mice (male: 298.3 vs. 291.4 pg/ml; female: 198.5 vs. 192.8 pg/mL; *p* > 0.05). In two-year-old mice, the IGF-1 levels were slightly higher in L-VC male mice compared with H-VC males (269.9 vs. 250.3 pg/mL; *p* > 0.05), and its levels were significantly higher in L-VC compared with H-VC female mice (245.9 vs. 127.3pg/mL; *p* < 0.05).

Furthermore, the regression analysis of IGF-1 values based on each individual animal, including all ages and genders regardless of diets, demonstrated that serum free IGF-1 levels were negatively correlated with serum LDL (*r* = 0.49; *p* < 0.01; Figure 2(e)).

## 4. Discussion

Our study documents the effects of a long-term intake of low and high levels of vitamin C in ‘humanized' mice of different ages on important metabolic parameters such as lipid and hormonal profiles. A ‘humanized' mouse model was defined based on its dependency on dietary vitamin C and an ability to produce human Lp(a) internally.

We observed that female mice had significantly higher serum and liver ascorbic acid levels than male mice consuming the same H-VC diet. This corresponds to findings by Kuo et al. that female adult Slc23a2+/− and Slc23a2+/+ mice had higher ascorbic acid levels in plasma and liver than in male mice. These researchers postulated that female mice had a lower vitamin C urinary secretion rate than male mice, which could lead to higher vitamin C concentrations in plasma and tissues expressing SVCT1, such as the liver [24].

Serum ascorbic acid levels lower than 10 uM in humans indicate scurvy. In our study, serum ascorbic acid concentrations in mice kept for 20 weeks on an L-VC diet ranged from 0 to 12 uM. Maintaining vitamin C at a low (scurvy) level for 20 weeks may have more pronounced metabolic impact by increased redox imbalance and higher stress on the endoplasmic reticulum, affecting protein synthesis [25], when compared with a four-to-six-week low-vitamin-C regimen applied in the majority of animal studies. Our earlier study has shown that a long-term insufficient vitamin C intake in Gulo (−/−) and Lp(a) mice negatively affects the brain aging process and promotes vascular deposition of Lp(a) [12].

As expected, we observed that serum LDL levels in L-VC male mice were significantly higher compared with those in H-VC mice, albeit only at the ages of eight to nine months and two years (at one year, both female and male mice in the L-VC group had lower serum LDL levels than H-VC mice). In our previous study on Gulo (−/−) and Lp(a) mice, we found a reverse relationship between serum ascorbic acid levels and serum total cholesterol and LDL levels after a six-week low-vitamin-C regimen [5]. The effects of vitamin C intake on lowering serum LDL have been demonstrated in guinea pigs, Gulo (−/−) mice, and humans [10, 26–31]. It has been shown that daily supplementation of 500 mg vitamin C is effective in reducing serum cholesterol and serum LDL levels in humans [32]. The mechanism of the observed relationship between vitamin C and LDL levels has not been clearly determined, but it has been reported that vitamin C deficiency inhibits the enzyme 7 *α*-hydroxylase. This can lead to reduced conversion from plasma cholesterol to bile acid, resulting in the accumulation of cholesterol in the serum [32, 33]. In addition, ascorbate decreases HMG-CoA reductase activity, the key enzyme in the cholesterol synthesis pathway [34].

The lower serum LDL levels we noted in both male and females aged one year in the L-VC group, compared with the H-VC group, may be an effect of mid-age-related metabolic changes in response to chronic very low and high intake of vitamin C. It has been shown that protein biosynthesis in mid-aged mice is affected by increased oxidative stress owing to vitamin C deficiency [25]. However, further investigation is needed to evaluate whether or not age-related metabolic differences and age-related cellular response to vitamin C deficiency plays a role in lipid homeostasis.

Further analysis revealed that serum LDL levels negatively correlated with serum free IGF-1 levels, which was statistically significant. Human studies have shown that increased levels of IGF-1 may reduce serum LDL and Lp(a) levels [35, 36]. One of the mechanisms involved might include IGF-1-mediated upregulation of cellular LDL update, which would result in decreased LDL levels in serum [37]. An earlier study also showed that continuous infusion of 100 ug/day IGF-1 to noncastrated female h-apo(a) transgenic mice led to a 2.5-fold decrease in plasma h-apo(a) levels and suggested that IGF-1 has independent effects in the transcription of the apo(a) gene [38]. In our study, the changes of free IGF-1 induced by vitamin C deficiency may play a role in regulating LDL and h-apo(a) metabolism. Lipid metabolism and hormone regulations are complex; the exact mechanics of the effects of vitamin C and IGF-1 on lipid homeostasis remain to be established.

Furthermore, our results showed a significantly elevated LDL/HDL ratio in male mice in the L-VC group at the age of 8-9 months and 2 years, indicating a less healthy lipid profile. However, the LDL/HDL ratio was not different in male mice aged one year and in female mice at all ages. These results suggest that lipid metabolism in young and old male mice is more affected by vitamin C deficiency than in mid-aged mature mice. During the 20 weeks of the experiment, we observed that L-VC mice aged 8-9 months and 2 years had weight loss and appeared moribund at the end of the experiment. The L-VC mice aged 8-9 months and 2 years had begun the 20 weeks of L-VC diet treatment when they were approximately 3-4 months old and 19-20 months old, respectively. The young mice just reached maturity from developmental stage and the aged mice have begun to show the senescent changes in all biomarkers [36]. The metabolic rate and metabolite differences [37] among young, mid-aged mature, and aged mice may play a role in their responses to stress.

Cellular metabolism surrounding growth hormone, IGF-1, and insulin is arguably the most studied in regard to linking the metabolic status and the pace of aging. Researchers have found that insulin resistance in humans increases with aging and declines in subjects older than 90 years [38, 39]. Indeed, long-lived subjects showed higher insulin sensitivity and a better preservation of beta-cell function than younger subjects did. However, the optimal IGF-1 levels for a long and healthy life are still unknown.

We observed that chronic vitamin C deficiency in young (8-9 months old) and old (2 years old) mice has more pronouncing effects on serum IGF-1 levels. In particular, 8-9-month-old mice displayed significantly lower serum IGF-1 levels compared with H-VC mice. Palka and colleagues found similar effects in scorbutic guinea pigs showing decreased serum IGF-1 levels by 25%–33% [40], and suggested that vitamin C plays a role in the regulation of animal growth. In our study, mice aged 8-9 months had begun the 20 weeks of L-VC diet when they were 3-4 months old, which is considered as young adults. During adolescence, it is important that the IGF-1 levels dramatically increase to contribute to the growth of organisms. Early-life IGF-1 deficiency not only negatively influences body growth and tissue development but also increases the risk of developing age-related pathology later in life [41, 42].

In contrast to young mice, we found that L-VC old mice (2 years old) had higher serum IGF-1 levels than H-VC mice. A previous study on the effects of vitamin C deficiency on insulin resistance in Gulo (−/−) mice showed that 12 weeks of vitamin-C-deficient diet (33 mg/L vitamin C) promoted insulin resistance [43]. Thus, the dysregulation of serum IGF-1 caused by vitamin C deficiency shown in our study may explain the increased insulin resistance in vitamin C deficient Gulo (−/−) mice presented in the previous report. Overall, both our study and the published data suggest that adequate vitamin C supplementation might be a protective measure to delay the onset of insulin resistance and its related metabolic syndromes.

As expected, we observed that serum testosterone in male mice and estradiol levels in female mice decreased with age. We found that the average serum testosterone levels decreased in L-VC mice at the age of 8-9 months and one year compared with H-VC mice, but the difference was not significant, which was most likely explained by the biological variations among the animals. However, the average serum testosterone levels in L-VC mice were approximately three- to fourfold lower in mice of an age of 8-9 months and 1 year compared with H-VC mice of corresponding age. A previous study also found a significant decrease of testosterone levels in male rats under scurvy conditions, compared with rats supplemented with 250 mg/kg and 400 mg/kg vitamin C, and the effect was dose dependent [44]. This may relate to the role of vitamin C in hydroxylating cholesterol in the sex steroid hormones pathway, resulting in the promotion of steroidogenesis [45].

## 5. Conclusions

In conclusion, our study shows that chronic vitamin C deficiency results in a negative impact on lipid metabolic profile, testosterone and estradiol levels, and IGF-1 regulations in Gulo (−/−) and Lp(a)+ mice throughout the aging process. Metabolic consequences of low vitamin C intake were especially observed at the young and old age. This suggests that long-term, chronic vitamin C deficiency may facilitate the development of age-related diseases. Our results further emphasize the importance of consistent adequate vitamin C intake throughout the lifetime to maintain normal hormonal balance together with a favorable lipid homeostasis, which are essential components for optimal health.

## Figures and Tables

**Table 1:** Serum levels of total cholesterol, low density lipoprotein (LDL), high density lipoproteins (HDL), triglycerides, and LDL/HDL ratio in each age group and gender. Data are expressed as mean ± SD. *n* = 3–6 mice per group. For Tukey's HSD tests, ^*∗*^represents a statistically significant difference between H-VC groups and L-VC groups of the same age and gender at the significance level of 0.05; ^*∗∗*^represents *p* < 0.01; ^#^represents a statistically significant difference between all three age groups of the same gender and diet at the significance level of 0.05.

Parameters (mg/mL) ± SD	Diet	*M*			*F*		
		8-9 months	1 year	2 years	8-9 months	1 year	2 years
Total cholesterol	H-VC	126.8 ± 14.6	140.5 ± 26.2	100.5 ± 6.2^#^	144.4 ± 7.2	158.7 ± 20.2	140.8 ± 16.2
	L-VC	127.3 ± 48.9	101.5 ± 13.0^*∗*^	149.5 ± 35.6^*∗*^	154.8 ± 19.5	124.5 ± 18.4^*∗*^	132.8 ± 7.1

Low density lipoprotein (LDL)	H-VC	21.7 ± 6.6	33.9 ± 18.2	13.3 ± 2.3^#^	64.2 ± 7.2	73.4 ± 15.2	65.4 ± 12.2
	L-VC	60.4 ± 7.4^*∗*^	13.1 ± 3.1^*∗*^	66.9 ± 31.9^*∗*^	69.5 ± 26.5	43.6 ± 18.7^*∗*^	59.1 ± 5.7

High density lipoprotein (HDL)	H-VC	86.5 ± 16.2	89.4 ± 16.3	60.0 ± 6.5^#^	62.6 ± 4.9	76.5 ± 6.7^#^	62.3 ± 6.1
	L-VC	42.3 ± 27.7^*∗*^	57.2 ± 8.9^*∗*^	55.5 ± 12.1	58.7 ± 0.4	50.3 ± 6.4^*∗*^	59.2 ± 5.8

Triglyceride	H-VC	172.6 ± 60.5	276.7 ± 60.4^#^	164.3 ± 29.6	142.4 ± 12.2	206.3 ± 42.0^#^	122.5 ± 21.8
	L-VC	153.6 ± 32.0	303.3 ± 29.6	255.3 ± 64.6^*∗*^	126.1 ± 32.6	245.1 ± 12.0	231.6 ± 99.4^*∗*^

LDL/HDL ratio	H-VC	0.3	0.4	0.2	1	1	1.1
	L-VC	1.1^*∗∗*^	0.2	1.2^*∗∗*^	1.2	0.9	1

## Data Availability

The data used to support the findings of this study are available from the corresponding author Aleksandra Niedzwiecki (author@drrath.com) upon request.
